# Shedding new light on the origins of olfactory neurons

**DOI:** 10.7554/eLife.00648

**Published:** 2013-03-26

**Authors:** Tanya T Whitfield

**Affiliations:** 1 **Tanya T Whitfield** is an *eLife* reviewing editor, and is at the MRC Centre for Developmental and Biomedical Genetics, Department of Biomedical Science, University of Sheffield, Sheffield, United Kingdomt.whitfield@sheffield.ac.uk

**Keywords:** developmental neurobiology, olfactory development, microvillous sensory neurons, neurogenesis, neural crest migration, Zebrafish

## Abstract

Sensory neurons in the nose of the zebrafish are derived from both neural crest cells and placode cells.

**Related research article** Saxena A, Peng BN, Bronner ME. 2013. Sox10-dependent neural crest origin of olfactory microvillous neurons in zebrafish. *eLife* **2**:e00336. doi: 10.7554/elife.00336

**Image** The microvillous sensory neurons (green) in the nose of a zebrafish are derived from neural crest cells

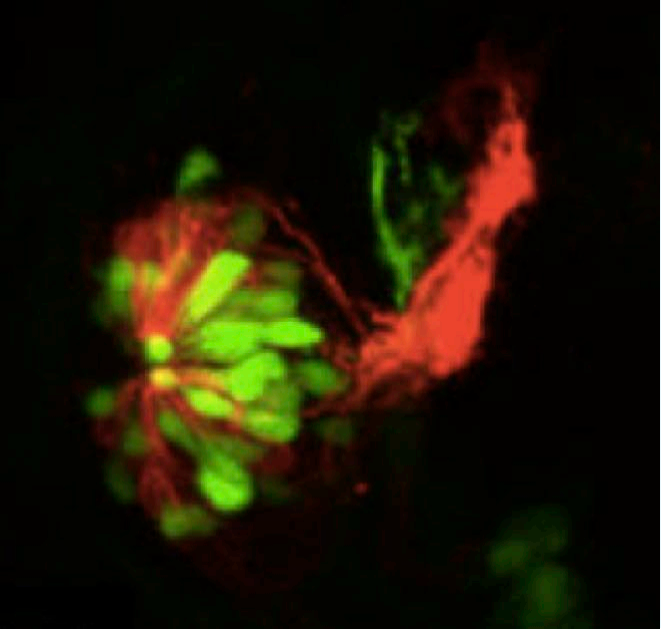

The nose of the five-day old zebrafish larva is deceptively compact and neat: a pair of epithelial vesicles tucked between the eye and the forebrain, distinct and separate from the surrounding tissue. This compactness, however, belies the fact that the olfactory system of the zebrafish has its origins in a surprisingly large region of the embryo. Vertebrate embryos contain two main types of cells that contribute to the formation of the peripheral sensory organs of the head—neural crest cells and placode cells (Figure 1A). In the zebrafish, embryonic cells converge from a wide region to form the olfactory placode (Whitlock and Westerfield, 2000), and the sensory neurons of the olfactory system were thought to be derived exclusively from these placode cells. However, the precise origin of all the different types of cells in the olfactory system has long been the subject of controversy and debate. Now, writing in *eLife*, Ankur Saxena, Brian Peng, and Marianne Bronner of the California Institute of Technology report that some olfactory sensory neurons are derived from neural crest cells rather than the placode (Saxena et al., 2013). Moreover, these neural crest cells migrate into the epithelial vesicles from even further afield than the placode cells do.

**Figure 1.**
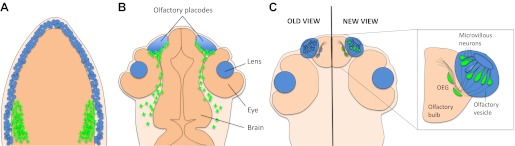
A new view of the origins of olfactory sensory neurons. (**A**) During the first day of development, both neural crest cells (green) and placode cells (blue) form around the edges of the developing nervous system. (**B**) By the end of the first day, the placode cells have converged to form the olfactory placodes and the lens of each eye. (Other derivatives of placode cells are not shown.) Neural crest cells now surround the placodes. (**C**) By the third day of development, the olfactory vesicle has formed, and sensory neurons project into the olfactory bulb. Previously it was thought that all sensory neurons and their supporting glia derived from the placode (left). However, Saxena and colleagues now show that many (but not all) of the microvillous neurons derive from neural crest cells (right). Supporting olfactory ensheathing glia (OEG) cells might also derive from neural crest cells, but this has not yet been shown for fish.

The zebrafish embryo is an ideal system in which to explore questions of cell origin because it is transparent, a feature that facilitates imaging studies, and because it develops rapidly. The approach adopted by the Caltech team is simple and non-invasive: choose a gene promoter that drives gene expression in the tissue of interest, hook it up to green fluorescent protein (GFP), and then watch where the cells labelled with GFP go. Saxena and colleagues used the *sox10* promoter to drive GFP expression in neural crest cells and then employed time-lapse confocal microscopy to follow these cells as they moved within the embryo. The neural crest cells migrated towards the olfactory placode during the first day of development, forming a capsule that surrounded the placode (Figure 1B). However, they did not mix with the cells in the placode to any significant extent. This is consistent with the results of a similar recent study by Kathleen Whitlock of the Universidad de Valparaíso and co-workers (Harden et al., 2012).

During the second day of development, however, some of the labelled neural crest cells moved out of the capsule surrounding the placode and into the olfactory epithelium (Figure 1C). Once there, they increased their production of both *sox10*:GFP and Sox10 protein, and formed a class of sensory neurons called microvillous neurons, identified by their position in the epithelium, characteristic tear-drop shape and appropriate molecular signature (Saxena et al., 2013). This result was a real surprise, as the classical view was that all olfactory sensory neurons derived from placode cells. To confirm that they were really following neural crest cells, Saxena et al. labelled the nuclei of small groups of neural crest cells with a photoconvertible protein that made their nuclei appear red when imaged by the confocal microscope. They found that the microvillous neurons (which look green because they contain GFP) had red nuclei, confirming that they had originated from neural crest cells. Moreover, they found that a different class of neurons, called ciliated sensory neurons, did not have red nuclei.

To provide extra evidence that microvillous neurons are derived from neural crest cells, Saxena et al. used laser ablation to destroy groups of neural crest cells labelled with GFP before they entered the nasal epithelium. As expected, this resulted in a depletion of microvillous neurons in the nose, but had only a minimal effect on the ciliated neurons. In addition to confirming that microvillous neurons are derived from neural crest cells, this also suggests that placode cells cannot compensate for the loss of sensory neurons derived from neural crest cells.

Finally, Saxena, Peng, and Bronner explored whether the transcription factor Sox10 was necessary for the development of microvillous neurons. They used a synthetic molecule called a morpholino to knock down *sox10* gene function during different stages of development. They found that the neural crest cells required Sox10 to enter the epithelium and to form the microvillous sensory neurons. However, the results of morpholino experiments are inherently variable, so it will be important to corroborate this finding by examining zebrafish that carry a mutation in *sox10*. Microvillous neurons should be depleted or missing from these mutants, whereas the ciliated neurons should not be affected.

Transgenic labelling techniques have also been used to show that various cell types in the olfactory systems of mice and chicks are derived from neural crest cells. The best evidence here supports a neural crest origin for olfactory ensheathing glial cells, which lie outside the olfactory epithelium (Barraud et al., 2010; Forni et al., 2011; Katoh et al., 2011; Suzuki et al., 2013). Although some of these studies found occasional labelled olfactory neurons, it is not yet clear if a neural crest origin for microvillous neurons is a general feature in all vertebrates, or if it is specific to fish. There are certainly some anatomical differences between fish and other vertebrates: zebrafish, for example, do not have a vomeronasal organ, which is the location of microvillous neurons in mammals. It will also be interesting to test whether a neural crest origin for olfactory ensheathing glia is conserved in fish, as has been suggested but not yet tested fully (Harden et al., 2012).

Recent studies, also using transgenic *sox10* constructs, have revealed additional previously unknown neural crest derivatives in the zebrafish and mouse (Simon et al., 2012; Mongera et al., 2013). These studies, and the work of Saxena and colleagues, all illustrate the remarkable developmental plasticity of the neural crest cell. Olfactory sensory neurons are capable of regeneration, and olfactory ensheathing glia can support new axon growth, even in the adult mammal. These cell types may offer exciting possibilities for patient-specific therapies to repair damaged nerves, for example. For the developmental biologist, the findings are a reminder that a single organ system is often assembled in the embryo from a diverse array of cell types, and they open up further questions on how migration and specification of the neural crest is controlled.

## References

[bib1] Barraud P Seferiadis AA Tyson LD Zwart MF Szabo-Rogers HL Ruhrberg C 2010 Neural crest origin of olfactory ensheathing glia. Proc Natl Acad Sci USA 107:21040–5 doi: 10.1073/pnas.1012248107 21078992 PMC3000254

[bib2] Forni PE Taylor-Burds C Melvin VS Williams T Wray S 2011 Neural crest and ectodermal cells intermix in the nasal placode to give rise to GnRH-1 neurons, sensory neurons, and olfactory ensheathing cells. J Neurosci 31:6915–27 doi: 10.1523/JNEUROSCI.6087-10.2011 21543621 PMC3101109

[bib3] Harden MV Pereiro L Ramialison M Wittbrodt J Prasad MK McCallion AS 2012 Close association of olfactory placode precursors and cranial neural crest cells does not predestine cell mixing. Dev Dyn 241:1143–54 doi: 10.1002/dvdy.23797 22539261 PMC4240535

[bib4] Katoh H Shibata S Fukuda K Sato M Satoh E Nagoshi N 2011 The dual origin of the peripheral olfactory system: placode and neural crest. Mol Brain 4:34 doi: 10.1186/1756-6606-4-34 21943152 PMC3215936

[bib5] Mongera A Singh AP Levesque MP Chen YY Konstantinidis P Nüsslein-Volhard C 2013 Genetic lineage labeling in zebrafish uncovers novel neural crest contributions to the head, including gill pillar cells. Development 140:916–25 doi: 10.1242/dev.091066 23362350

[bib6] Saxena A Peng BN Bronner ME 2013 Sox10-dependent neural crest origin of olfactory microvillous neurons in zebrafish. eLife 2:e00336 doi: 10.7554/elife.00336 PMC3601810 23539289

[bib7] Simon C Lickert H Götz M Dimou L 2012 Sox10-iCreERT2: a mouse line to inducibly trace the neural crest and oligodendrocyte lineage. Genesis 50:506–15 doi: 10.1002/dvg.22003 22173870

[bib8] Suzuki J Yoshizaki K Kobayashi T Osumi N 2013 Neural crest-derived horizontal basal cells as tissue stem cells in the adult olfactory epithelium. Neurosci Res 75:112–20 doi: 10.1016/j.neures.2012.11.005 23228673

[bib9] Whitlock KE Westerfield M 2000 The olfactory placodes of the zebrafish form by convergence of cellular fields at the edge of the neural plate. Development 127:3645–53 10934010 10.1242/dev.127.17.3645

